# Preoperative Subconjunctival Injection of Mitomycin C Versus Intraoperative Topical Application as an Adjunctive Treatment for Surgical Removal of Primary Pterygium

**DOI:** 10.4103/0974-9233.75883

**Published:** 2011

**Authors:** Ehab M. Ghoneim, Ahmed A. Abd-El Ghny, Amro A. Gab-Allah, Mohamed Z. Kamal

**Affiliations:** Department of Ophthalmology, Faculty of Medicine, Suez Canal University, Ismailia, Egypt

**Keywords:** Bare Sclera Technique, Mitomycin C, Pterygium, Scleral Thinning

## Abstract

**Purpose:**

To compare the efficacy of preoperative local injection of mitomycin C (MMC) to intraoperative application of MMC in the prevention of pterygium recurrence after surgical removal.

**Materials and Methods:**

Seventy eyes of 70 patients with primary pterygia were randomly allocated to two groups. The first group (Group A, 35 eyes) received 0.1 ml of 0.15 mg/ml of subconjunctival MMC injected into the head of the pterygium 24 h before surgical excision with the bare sclera technique. The second group (Group B 35 eyes) underwent surgical removal with the bare sclera technique with intraoperative application of MMC (0.15 mg/ml) over bare sclera for 3 min. The study was performed between March 2007 and December 2008, and follow up was performed for 1 year postoperatively. Differences between frequencies in both groups were compared by the Chi-square test or Fisher exact test. Differences between means in both groups were compared by Student’s t-test. P < 0.05 was considered significant.

**Results:**

The rate of pterygium recurrence was 5.70% in Group A and 8.57% in Group B at 1 year postoperatively (P>0.05). Postoperatively, scleral thinning occurred in one eye in each group that resolved by 5 months postoperatively. No serious postoperative complications occurred in either group.

**Conclusion:**

Preoperative local injection of 0.15 mg/ml MMC is as effective as intraoperative topical application of 0.15 mg/ml MMC for preventing pterygium recurrence after surgical removal.

## INTRODUCTION

Pterygium is a common ocular health problem in Egypt due to the hot climate and exposure to ultraviolet light. In our experience, simple excision of the pterygium has a very high rate of recurrence of approximately 30-50%. Adjunctive treatments, including radiation, antimetabolites, conjunctival grafts and limbal grafts, are used to decrease the rate of recurrence after surgical excision.^1^ Adjunctive mitomycin C (MMC) has become a more commonly used technique in preventing pterygium recurrence. The mechanism of action of MMC in the prevention of pterygium recurrence is the inhibition of episcleral fibroblast proliferation.^2^,^3^

Long term use of topical MMC eye drops after pterygium surgery can cause serious complications such as secondary glaucoma, corneal edema, scleral necrosis, and sudden onset mature cataract.^4^ Hence, MMC use has been limited to use as a single intraoperative application with high success rates and fewer complications.

Alternately, subconjunctival injection of MMC can be used as adjunctive therapy before pterygium excision as it allows exact titration of MMC delivery to the activated fibroblasts and minimizes epithelial toxicity.^5^ For example, Donnenfeld *et al*.^5^ reported a success rate of 94% with subconjunctival injection of MMC, 1 month prior to pterygium excision with the bare sclera technique.

In the current study, we compare the efficacy of preoperative local injection of mitomycin C (MMC) to intraoperative application to prevent the recurrence of pterygium after surgical removal.

The aim of this study was to reduce the exposure time of subconjunctival tissues to MMC 24 h before excision in an attempt to avoid complications related to MMC.

## MATERIALS AND METHODS

From march 2007 and December 2008, 70 eyes of 70 subjects with primary pterygia were randomly assigned to undergo surgical removal of pterygium into one of two groups: Group A (35 eyes) received subconjunctival MMC 24 h before pterygium excision with the bare sclera technique; Group B (35 eyes) underwent pterygium removal with the bare sclera technique and intraoperative application of MMC. Randomization was performed with random number tables. Informed consent was obtained from all subjects enrolled in the study. The protocol of the study was approved by the ethics committee in the faculty of Medicine.

Individuals were considered eligible for treatment if they had a primary fleshy pterygium. Individuals with pterygium associated with dry eye, uveitis, glaucoma, and chronic blepharitis resistant to treatment were excluded from the study due to a high risk of MMC toxicity.

### Surgical technique for Group A

Subjects received a subconjunctival mitomycin C (0.1 ml of 0.15 mg/ml) 24 h preoperatively with the following technique. Two drops of benoxinate hydrochloride 0.5% topical anesthetic were instilled in the operative eye. A cotton swab soaked in benoxinate hydrochloride 0.5% then was directly applied to the pterygium for 3 min to obtain topical anesthesia. Under an operating microscope, subconjunctival injection was performed with a 30-gauge needle and an insulin syringe containing 0.1 ml of 0.15 mg/ml MMC. The injection site was directly into the pterygium at the limbus. After injection, the conjunctival sac was irrigated with saline to wash out excess MMC and the patient received one drop of ciprofloxacin 0.3%, with instructions to continue use four times daily.

Twenty-four hours after MMC injection, the patients underwent the bare sclera excision of the pterygium. A cotton stick soaked in benoxinate hydrochloride 0.5% was applied directly to the pterygium head for 3 min to obtain topical anesthesia. Subsequently, 0.5 ml of 2% Lidocaine was injected subconjunctivally with a 30-gauge insulin syringe. The pterygium was grasped at the limbus with 0.3-mm toothed forceps, and the head of the pterygium was dissected from the underlying cornea. The pterygium was then grasped, and the underlying conjunctiva and Tenon’s capsule were dissected to bare sclera approximately 4 mm posterior to the limbus. The scleral bed and the cornea were then polished with a number 15 surgical blade and the scleral bed was cauterized.^6^

### Surgical technique for Group B

Eyes underwent pterygium excision with the bare sclera technique described above. After cauterization of the scleral bed, a microsponge soaked in MMC 0.15 mg/ml was applied to bare sclera for 3 min. The conjunctival sac was irrigated with saline after removal of the microsponge to wash out excess MMC.

The postoperative treatment regime was the same in both groups: topical eye ointment (tobramycin and dexamethasone combination) was applied; the eyes were patched until day 1 postoperatively. Postoperatively, patients were treated with a combination of tobramycin and dexamethasone eye drops four times daily for 1 week, followed by a tapering schedule for the following 3 weeks. Eye ointment (tobramycin and dexamethasone combination) was applied for 2 weeks at bed time.

Patients were followed up at 1 day, 1 week, 1 month, 3 months, 6 months and 1 year after pterygium excision. Patients were examined at all visits for conjunctival erythema, epithelial defects, and pterygium recurrence. Recurrence was defined as fibrovascular growth of conjunctival tissue crossing at least 1 mm past the corneoscleral limbus.

Data were imported into Statistical Package for the Social Sciences (SPSS version 10.0) software for analysis. Baseline characteristics of the study population were presented as frequencies and percentages (%) or mean values and standard deviations (SD). Sex, right or left eye, success and recurrence rates and complications were treated as categorized variables. Age and intraocular pressure (IOP) were treated as continuous variables. Differences between frequencies in both groups were compared by the chi-square test when all expected values in a 2×2 table > 5 or the Fisher exact test when one of the expected values in the 2×2 table < 5. Differences between means in both groups were compared by Student’s t-test. A *P* value of < 0.05 was considered significant.

## RESULTS

The study cohort was composed of 70 eyes of 70 subjects divided into two groups of 35 eyes each with primary fleshy pterygia. The mean age was 33.0±7.14 years (range 26 to 50 years) for Group A and 34.0 ±8.17 years (range 27 to 51 years) for Group B. Table 1 presents the preoperative demographic data for the entire cohort.

**Table 1:** Preoperative demographic data for both groups

	Sex				Eye			
	Male		Female		Right		Left	
Group A	15	42.9%	20	57.1%	17	48.6%	18	51.4%
Group B	16	45.7%	19	54.3%	19	54.3%	16	45.7%

Group A denotes subjects who underwent preoperative local injection of 0.15 mg/ml mitomycin C, Group B denotes subjects who underwent intraoperative topical application of 0.15 mg/ml mitomycin C

**Figure 1:**
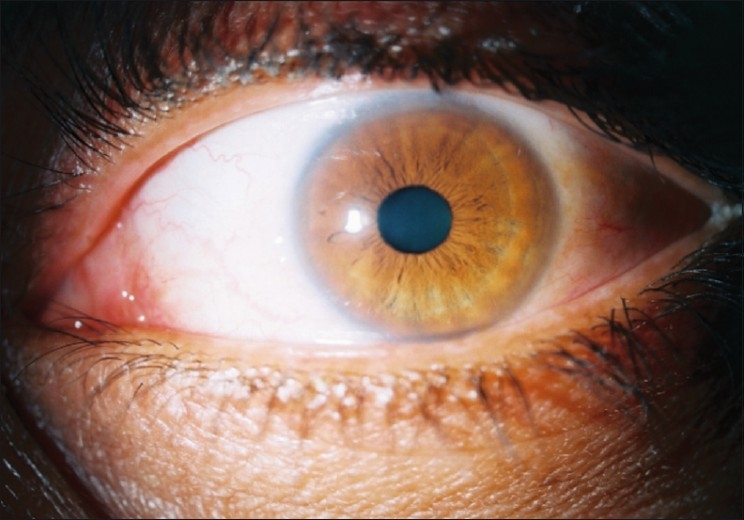
Left eye of a subject with no signs of recurrence 10 months after pterygium removal with the bare sclera technique and preoperative local injection of mitomycin C (Group A)

All subjects were seen on the first postoperative day and had epithelial and conjunctival defects. By the first week postoperative visit, all epithelial defects were completely closed. At 1 year postoperatively, the recurrence rate was 5.7% (2 eyes) in Group A and 8.57% (3 eyes) in Group B (*P*=0.99) [Table 2]. The pterygia recurred between the fourth and seventh postoperative months in these cases. Figure 1 presents the left eye of a Group A subject with no signs of recurrence 10 months after pterygium removal.

**Table 2:** Success rate and recurrence rate in both groups

	Group A	Group A	*P*-value
Success rate	94.3% (33 eyes)	91.43% (32 eyes)	0.99
Recurrence rate	5.7% (2 eyes)	8.57% (3 eyes)	

*P* value <0.05 was considered statistically significant, Group A denotes subjects who underwent preoperative local injection of 0.15 mg/ml mitomycin C, Group B denotes subjects who underwent intraoperative topical application of 0.15 mg/ml mitomycin C

The mean preoperative IOP was 13.47±1.53 mmHg in Group A and 13.05 ±1.18 mmHg in Group B (*P*>0.05). The mean IOP 1 year postoperatively was 12.73± 1.08 mmHg in Group A and 13.01±1.01 mmHg in Group B (*P*>0.05).

Complications were minored in both groups [Table 3]. Conjunctival irritation occurred in 14 eyes in Group A and 11 eyes in Group B with all cases resolving within the first 2 postoperative weeks [Table 3]. Subconjunctival hemorrhage occurred in seven eyes in Group A and eight eyes in Group B and resolved in all cases after the third postoperative week [Table 3].

**Table 3:** Postoperative complications in eyes that received preoperative or intraoperative mitomycin C for pterygium surgery

Complications	Group A		Group B		*P*-value
	No.	%	No.	%	
Conjunctival irritation	14	40	11	27.5	0.45
Subconjunctival hemorrhage	7	20	8	12.5	0.78
Scleral thinning	1	2.9	1	2.9	0.99
Superficial punctuate keratits	0	0	1	2.9	0.99
Cataract formation	0	0	0	0	1.00
Corneal thinning	0	0	0	0	1.00
Photophobia	8	22.9	10	28.6	0.58

*P* value <0.05 was considered statistically significant, Group A denotes subjects who underwent preoperative local injection of 0.15 mg/ml mitomycin C, Group B denotes subjects who underwent intraoperative topical application of 0.15 mg/ml mitomycin C

**Figure 2:**
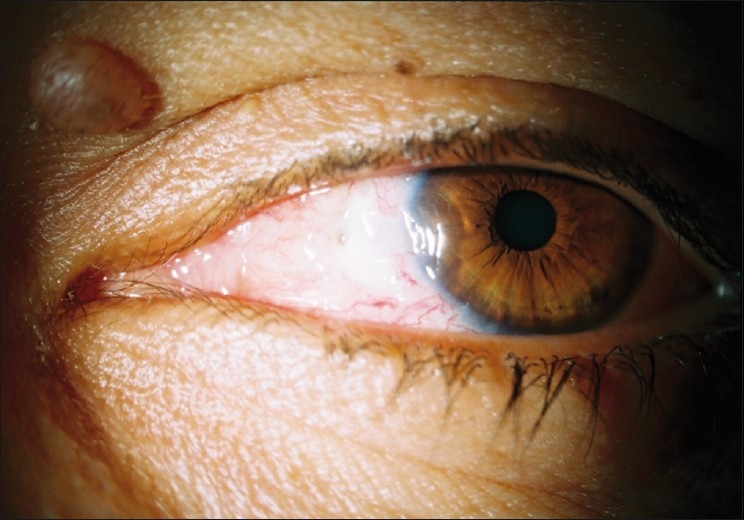
Mild scleral thinning in the left eye of the patient in Group B 2 months after pterygium surgery

Eight subjects complained of photophobia in Group A and 10 subjects in Group B [Table 3]. By 2 weeks postoperatively photophobia had completely resolved in all subjects in the cohort. Punctuate epithelial keratopathy was observed in one eye in Group B that resolved after 2 weeks postoperatively. Scleral thinning was observed 2 months postoperatively, occurring in one eye in Group A and one eye in Group B [Figure 2]. All subjects were treated with artificial tears drops and topical ointment and the condition improved after 3 months of treatment.

Complications such as corneal ulceration or perforation, scleral perforation, secondary glaucoma, and sudden onset mature cataract did not occur for the duration of this study.

## DISCUSSION

The mechanism of action of MMC in the prevention of pterygium recurrence has been attributed to the inhibition of fibroblast proliferation of the episclera.^7^,^8^ MMC has a prolonged, if not permanent effect of suppressing human fibroblasts.^7^,^8^ This prevents the development of fibrosis and aggressive wound healing that is responsible for pterygia recurrence. Adjunctive MMC for pterygium surgery was first described by Kunitomo and Mori in Japan in 1963.^9^

In an attempt to decrease ocular morbidity, the intraoperative use of MMC applied directly to the scleral bed has gained increasing acceptance. In this technique, after bare sclera excision, 0.2 to 0.4 mg/ml MMC is applied directly to the scleral bed for 2 to 5 min.^10^ The advantages of this technique include decreasing the MMC dosage, the use of MMC only in the operating room and application of MMC directly to the area of pathology rather than to the entire ocular surface.^11^

Donnenfeld *et al*,^5^ evaluated the safety and efficacy of subconjunctival 0.15 mg/ml MMC as adjunctive therapy before pterygium surgery and reported a high success rate.^5^ Donnenfeld *et al*, 5 performed subconjunctival injection of MMC 1 month before pterygium surgery while in our study MMC was injected into the subconjunctival space 24 h before pterygium surgery. MMC is a very potent antifibrotic agent therefore only short exposure time is required. To our knowledge this is the first peer review publication of MMC injection into the subconjunctival space 24 h before pterygium surgery. In our study, every attempt was made to reduce the concentration of MMC for use in pterygium surgery to minimize the possible side effects, yet maintain the least possible effective dose. Chen *et al*,^8^ reported that a concentration of 0.10 mg/ml MMC inhibits fibroblast replication, while concentrations of 0.3 mg/ml cause fibroblast cell death. We chose a concentration of 0.15 mg/ml as this concentration was slightly higher than the therapeutic window but well below the toxic level associated with cell death.

In the current study we found that both preoperative use and intraoperative use of MMC were equally successful for pterygium surgery. For example, the rate of recurrence was 5.7% (2 eyes) in Group A and 8.57% (3 eyes) in Group B at 1 year postoperatively. In previous literature the rate of recurrence after surgical removal of pterygium with the bare sclera technique alone ranges from 35 to 70%. We did not include a control group of subjects undergoing the bare sclera technique without MMC due to this high recurrence rate. To include such a control group would result in greater ocular morbidity and prolonged medical therapy.

The rate of recurrence of 5.7% in Group A concurs with Donnenfeld *et al*,^5^ rate of 6% who used the same technique; however they performed subconjunctival injection 1 month prior to pterygium surgery. In Group B where MMC was used intraoperatively in a concentration of 0.15 mg/ml over bare sclera for 3 min, the rate of recurrence was 8.57% (three eyes). This concurs with previous studies on intraoperative application of MMC with a rate of recurrence of 5% and 6%.^10^,^11^ The concentration used in the previous studies^10^ was 0.2 mg/ml while in our study the concentration was lower (0.15 mg/ml). The lower concentration in our study had comparable efficacy along with the added advantage of reducing possible toxicity to the ocular tissues.

In the current study most postoperative complications were mild such as subconjunctival hemorrhage, photophobia and conjunctival irritation, all of which were related to the surgical manipulation. Complications of MMC use included only one eye (2.9%) from Group A and one eye (2.9%) in Group B developing mild scleral thinning 2 months postoperatively which was successfully treated with topical artificial tears (drops and ointment) and withdrawal of corticosteroid and antibiotic combination drops. Follow up of these eyes was performed until recovery was complete at the end of the fifth month postoperatively. The two cases of scleral thinning were similar to ones documented by Carrasco *et al*,^12^ which were likely due to bad patient selection as they may have had dry eye syndrome. By investigating our cases of scleral thinning, we found that the patients used corticosteroids more frequently than recommended-instilling drops every time their eyes felt irritated; this frequent administration of steroids postoperatively delayed wound healing and induced scleral thinning.

Despite being well-documented^4^ complications of ocular MMC application, corneal ulcerations and perforations, scleral perforation, cataract formation, anterior uveitis, and IOP (secondary glaucoma) did not occur in our study.

The adjunctive use of MMC with surgery for primary pterygia with both techniques, either preoperative subconjunctival injection (Group A) or intraoperative MMC application (Group B), was equally effective. We prefer our techniques over the use of exogenous tissue (amniotic membrane transplantation and human processed pericardium) because of decreased surgical intervention, more rapid healing time, less patient discomfort, and decreased cost.^13^–^14^ Additionally, the technique is less time consuming than conjunctival graft and does not interfere with the success of glaucoma surgery in the future.

In conclusion, preoperative local injection of MMC 0.15 mg/ml is as effective as intraoperative topical application of MMC 0.15 mg/ml for prevention of the recurrence of pterygium after surgical removal with the bare sclera technique.
